# Comprehensive Analysis of mTORC1 Signaling Pathway–Related Genes in the Prognosis of HNSCC and the Response to Chemotherapy and Immunotherapy

**DOI:** 10.3389/fmolb.2022.792482

**Published:** 2022-04-29

**Authors:** Zhao Ding, Hailong Shen, Ke Xu, Yu Wu, Shuhao Wang, Fangzheng Yi, Daming Wang, Yehai Liu

**Affiliations:** ^1^ Department of Otorhinolaryngology-Head and Neck Surgery, The First Affiliated Hospital of Anhui Medical University, Hefei, China; ^2^ Anhui Medical University, Hefei, China; ^3^ Graduate School of Anhui Medical University, Hefei, China; ^4^ Department of Otolaryngology, General Hospital of Anhui Wanbei Coal Power Group, Suzhou, China

**Keywords:** signature, HNSCC, mTORC1, prognosis, nomogram

## Abstract

**Objective:** The mammalian target of the rapamycin complex 1 (mTORC1) signaling pathway has emerged as a crucial player in the oncogenesis and development of head and neck squamous cell carcinoma (HNSCC), however, to date, no relevant gene signature has been identified. Therefore, we aimed to construct a novel gene signature based on the mTORC1 pathway for predicting the outcomes of patients with HNSCC and their response to treatment.

**Methods:** The gene expression and clinical data were retrieved from The Cancer Genome Atlas (TCGA) and Gene Expression Omnibus (GEO) databases. The key prognostic genes associated with the mTORC1 pathway were screened by univariate Cox regression analyses. A prognostic signature was then established based on significant factors identified in the multivariate Cox regression analysis. The performance of the multigene signature was evaluated by the Kaplan–Meier (K–M) survival analysis and receiver operating characteristic (ROC) analysis. Based on the median risk score, patients were categorized into high- and low-risk groups. Subsequently, a hybrid prognostic nomogram was constructed and estimated by a calibration plot and decision curve analysis. Furthermore, immune cell infiltration and therapeutic responses were compared between the two risk groups. Finally, we measured the expression levels of seven genes by quantitative real-time polymerase chain reaction (qRT-PCR) and immunohistochemistry (IHC).

**Results:** The mTORC1 pathway–based signature was constructed using the seven identified genes (SEC11A, CYB5B, HPRT1, SLC2A3, SC5D, CORO1A, and PIK3R3). Patients in the high-risk group exhibited a lower overall survival (OS) rate than those in the low-risk group in both datasets. Through the univariate and multivariate Cox regression analyses, this gene signature was confirmed to be an independent prognostic risk factor for HNSCC. The constructed nomogram based on age, American Joint Committee on Cancer (AJCC) stage, and the risk score exhibited satisfactory performance in predicting the OS. In addition, immune cell infiltration and chemotherapeutic and immunotherapeutic responses differed significantly between the two risk groups. The expression levels of SEC11A and CYB5B were higher in HNSCC tissues than in normal tissues.

**Conclusion:** Our study established and verified an mTORC1 signaling pathway–related gene signature that could be used as a novel prognostic factor for HNSCC.

## Introduction

Head and neck squamous cell carcinoma (HNSCC) is the sixth most common cancer worldwide, originating from epithelial cells in various areas, including, but not limited to, the nasopharynx, oropharynx, hypopharynx, oral cavity, and larynx (Jemal et al., 2011). HNSCC is an often-fatal malignancy that is responsible for 431,000 annual deaths, with more than 835,000 new cases diagnosed every year (Bray et al., 2018). Because no effective screening method presently exists for identifying cases in the early stages, more than 60% of patients with HNSCC have already progressed to an advanced stage (stage III or IV) at the time of diagnosis (Machiels et al., 2020). Treatment strategies for early-stage (stage I/II) HNSCC include surgery or radiotherapy alone, whereas advanced-stage diseases require a multidisciplinary treatment strategy that combines surgical resection, radiotherapy, and chemotherapy (Tijink et al., 2006). The 5-year overall survival (OS) rate of patients with HNSCC is approximately 60% because of recurrence and metastasis, although there have been great advances in the therapeutic strategies for treating HNSCC in recent years, including immunotherapy and targeted molecular therapy (Ferris, 2015; Polverini et al., 2018). Therefore, developing a prognostic model capable of predicting clinical end points is of great importance for guiding clinical decisions and improving the understanding of the pathophysiology of the disease.

Many studies have investigated the relationship between the expression of various biomarkers and HNSCC prognosis. For example, the overexpression of CD44, a transmembrane glycoprotein that acts as a cell-surface adhesion receptor, has been used as a prognostic marker in laryngeal and pharyngeal cancer and as a target of therapeutic strategies (Kokko et al., 2011). Aldehyde dehydrogenase 1 (ALDH1) belongs to a family of intracellular enzymes that are involved in cellular detoxification and mechanisms driving multidrug resistance. High levels of ALDH1 in patient samples were shown to be associated with drug resistance and poor prognosis in HNSCC (Yu and Cirillo, 2020). However, HNSCC cells are characterized by a high degree of genomic instability, and polygenic changes may be required to drive cancer progression and tumor metastasis (Califano et al., 1996). Therefore, compared with a single-gene biomarker, a multigene signature model would be expected to exhibit a better predictive value, and researchers have recently built some multigene signatures for predicting clinical outcomes and guiding the treatment of those with HNSCC. For instance, Liu et al. (2021) constructed a new prognostic signature based on six genes that was capable of predicting both the responses to immunological treatment and prognosis of patients with HNSCC. In addition, other studies have shown that a constructed gene signature based on seven ferroptosis-related genes can be used to predict the prognosis of those with HNSCC (He et al., 2021).

The mammalian target of rapamycin (mTOR) is a serine/threonine kinase that binds to different proteins and assembles into two complexes: mTOR complex 1 (mTORC1) and mTOR complex 2 (mTORC2) (Mossmann et al., 2018). The mTOR pathway governs the processes that mediate cell metabolism, proliferation, survival, immune function, and apoptosis, and the pathway plays a vital role in the pathophysiology of diverse malignant tumors (Caro-Vegas et al., 2019). Many studies have been implemented to explore how the mTOR pathway modulates angiogenesis and tumor immunity (Bazzichetto et al., 2020). Many previous studies have shown that mTORC1 is tightly related to cancer progression. The activation of mTORC1 phosphorylates the eukaryotic initiation factor 4E-binding protein 1 (4E-BP1) and the p70 ribosomal S6 kinase 1 (S6K1), affecting mRNA transcription and protein translation (Meric-Bernstam and Gonzalez-Angulo, 2009). Furthermore, mTORC1 regulates autophagy through ULK-complex phosphorylation (Saxton and Sabatini, 2017) and plays an important role in regulating apoptosis (Shao et al., 2017). So, a signature based on the mTORC1 signaling pathway established to predict the prognosis of HNSCC patients would have important clinical applications.

In this study, we constructed prognostic models using univariate and multivariate Cox regression analyses based on data from The Cancer Genome Atlas (TCGA) cohort. The performance of the gene signature was validated using the Gene Expression Omnibus (GEO) dataset. We also developed a nomogram that can be applied in clinical practice. Most importantly, our signature is capable of distinguishing patients with HNSCC who are likely to respond to chemotherapy and immunotherapy.

## Materials and Methods

### Data Source and Processing

The gene expression profiles of patients with HNSCC, somatic mutations, and clinical data related to age, sex, stage, and follow-up time were downloaded from TCGA database on 10 July 2021. The data from patients with HNSCC in TCGA database were used as a training set to build the predictive gene signature. For further external validation, we used data from the GEO: GSE41613 cohort as a test group to certify the performance of the signature. Detailed clinical data for patients in TCGA and GEO cohorts are shown in Table 1. Related genes of the mTORC1 signaling pathway were collected from the Molecular Signatures Database (HALLMARK_MTORC1_SIGNALING). Gene Set Enrichment Analysis (GSEA) was used to assess whether mTORC1 signaling pathway–related genes were specifically enriched in the cancer tissues of patients with HNSCC.

**TABLE 1:** Clinical data of patients in the TCGA and the GEO validation cohort.

Variables	Subgroups	TCGA (N = 502)	Variables	Subgroups	GEO (N = 97)
Age	< 60	221	Age	< 60	50
	≥ 60	280		≥ 60	47
	NA	1	Gender	Female	31
Gender	Female	134		Male	66
	Male	368	Stage	I-II	41
Stage	I	19		III-IV	56
	II	95			
	III	102			
	IV	272			
	NA	14			
Grade	I	62			
	II	300			
	III	119			
	IV	2			
	NA	19			

### Construction and Verification of the mTORC1 Signaling Pathway–Related Gene Signature

First, the expression matrix of all the genes involved in the mTORC1 pathway was extracted from TCGA dataset. Subsequently, a univariate Cox regression analysis was performed to screen for potential genes that were significantly associated with the prognosis of patients with HNSCC (*p* < 0.01). Subsequently, a multivariate Cox regression analysis was conducted to further screen for the key genes that could be used in the construction of the model. Based on the model gene coefficients and expressions calculated from the results of the multivariate regression analysis, an equation was constructed to calculate the risk score for each individual patient using the following formula:

$$\text{Risk}\;\text{score}=\sum_{i=1}^{n}\text{Coef}\;{\text{i}}^{*}\text{Expr}\;\text{i},$$ (1)

where Coef i is the coefficient of gene i and Expr i represents the expression value of gene i. Based on this signature, a risk score was calculated for each patient in TCGA cohort and from this score, each patient was categorized into a high-risk or low-risk group, with the threshold being the median risk score. To further explore the prognostic value of the signature, the Kaplan–Meier (K–M) survival analysis and the log-rank test were utilized to investigate differences in survival between the high- and low-risk patients using the “survival” package in R statistical analysis software. Using the “timeROC” package in R, time-dependent receiver operating characteristic (ROC) curves were generated to investigate the reliability of the gene signature for predicting the prognosis. To further evaluate the robustness of the results, the risk model was validated using data from the GEO cohort.

### Analysis of Differences and Mutations in Genes Related to Prognosis

The differences in the expression of 25 genes related to prognosis in normal and tumor tissues were statistically analyzed using the Wilcoxon test. Copy number variation (CNV) is the most frequent type of genetic variation occurring in cancers and is associated with the occurrence and progression of tumors. Thus, we computed the frequencies of copy number gains or losses using the Genomic Identification of Significant Targets in Cancer (GISTIC) algorithm (Mermel et al., 2011). The distributions of CNVs in 25 chromosomes were plotted using the “RCircos” package in R software. A waterfall plot of the mutational landscape was generated using the “maftools” package.

### Independent Prognostic Analysis, Nomogram Construction, and Performance Assessment

Univariate and multivariate Cox regression analyses were performed on the data related to the signature and other clinical characteristics, including age, sex, and the American Joint Committee on Cancer (AJCC) stage in the training and test groups to clarify whether the risk score independently predicted clinical outcomes in patients with HNSCC. Parameters including the hazard ratio (HR), 95% confidence intervals (CIs), and *p* values were calculated using the “survival” package and visualized with the help of the “forestplot” package in R. The variables in the multivariate Cox regression analysis that independently predicted patient prognosis were used to construct the nomogram. Furthermore, the calibration plot was used to evaluate the calibration of the nomogram, and a decision curve analysis (DCA) was conducted to assess the clinical practicability of the nomogram by determining the net benefits at various threshold probabilities.

### Exploration of Tumor-Infiltrating Immune Cells

An immune infiltration analysis was subsequently conducted to evaluate the association between immune cell characteristics and the risk score. We compared the infiltration status of immunocytes between the high- and low-risk groups by using presently acknowledged algorithms, including TIMER (Li et al., 2020), CIBERSORT (Chen et al., 2018), CIBERSORT-ABS (Tamminga et al., 2020), QUANTISEQ (Plattner et al., 2020), MCPCOUNTER (Dienstmann et al., 2019), XCELL (Aran, 2020), and EPIC (Racle et al., 2017). The differences in tumor-infiltrating immune cells between the risk groups identified by these methods were analyzed using the Wilcoxon test, and the results were visualized with a heatmap. The R “ggplot2” package was used to complete the process.

### Prediction of Treatment Responses to Chemotherapy and Immunotherapy

This study compared the sensitivity of patients in the low- and high-risk groups to five chemotherapeutic agents commonly used in clinical practice (cisplatin, docetaxel, lapatinib, methotrexate, and paclitaxel). Immune checkpoint inhibitor drugs have revolutionized the treatment of multiple cancers, and the TIDE algorithm (http://tide.dfci.harvard.edu/) and subclass mapping (SubMap; https://cloud.genepattern.org/gp/) were used to predict the treatment responses to anti–programmed cell death 1 (PD1) and anti-cytotoxic T lymphocyte–associated protein 4 (CTLA4) antibodies (Wang et al., 2020a).

### Collection of Tissue Specimens

An experienced pathologist acquired fresh HNSCC tissues and adjacent normal tissues from patients treated at the First Affiliated Hospital of Anhui Medical University, none of whom received anticancer treatments before surgery. The collected tissue samples were stored at −80°C until use. All patients signed informed consent forms, and the study was approved by the Ethics Committee of the First Affiliated Hospital of Anhui Medical University.

### qRT-PCR and IHC

The TRIzol method was used to extract RNA from the tissue samples. The isolated RNA was used as a template for reverse transcription reactions using a RevertAid First-Strand cDNA Synthesis Kit (Thermo Fisher Scientific). A qRT-PCR analysis was performed using SYBR^®^ Premix Ex TaqTM Ⅱ (TaKaRa) and a Real-Time System (Roche Life Science). The primer sequences used are shown in Supplementary Table S1. Glyceraldehyde 3-phosphate dehydrogenase (GAPDH) was used as an internal reference. IHC was performed on formalin-fixed, paraffin-embedded specimens to quantify the levels of proteins encoded by the signature genes in cancerous and para-cancerous tissues. The HNSCC tissue sections were first deparaffinized in xylene and then dehydrated in ethanol. Antigen retrieval was performed using a citrate buffer (pH 6), followed by blocking with bovine serum albumin (BSA) for 1 h to prevent nonspecific binding of antibodies. The samples were then incubated in solutions containing specific primary antibodies (SEC11A, 1:150, Thermo Fisher Scientific; CYB5B, 1:100, Proteintech) followed by incubation in solutions containing secondary antibodies. Finally, the sections were visualized after staining with 3,3′-diaminobenzidine (DAB).

### Statistical Analysis

Log2 conversion was performed on the expression values of the genes used for the differential expression analysis in TCGA dataset. The “survminer” and “survival” packages in R software were used to depict the survival curves of the model genes in TCGA cohort, and the log-rank test was used to test for statistical differences. *P* < 0.05 was considered statistically significant, unless stated otherwise in the text. All analyses and the generation of graphical representations were performed using R version 3.6.3 and the corresponding packages described earlier.

## Results

### The mTORC1 Gene Set Significantly Differs Between HNSCC and Normal Tissues

**FIGURE 1:**
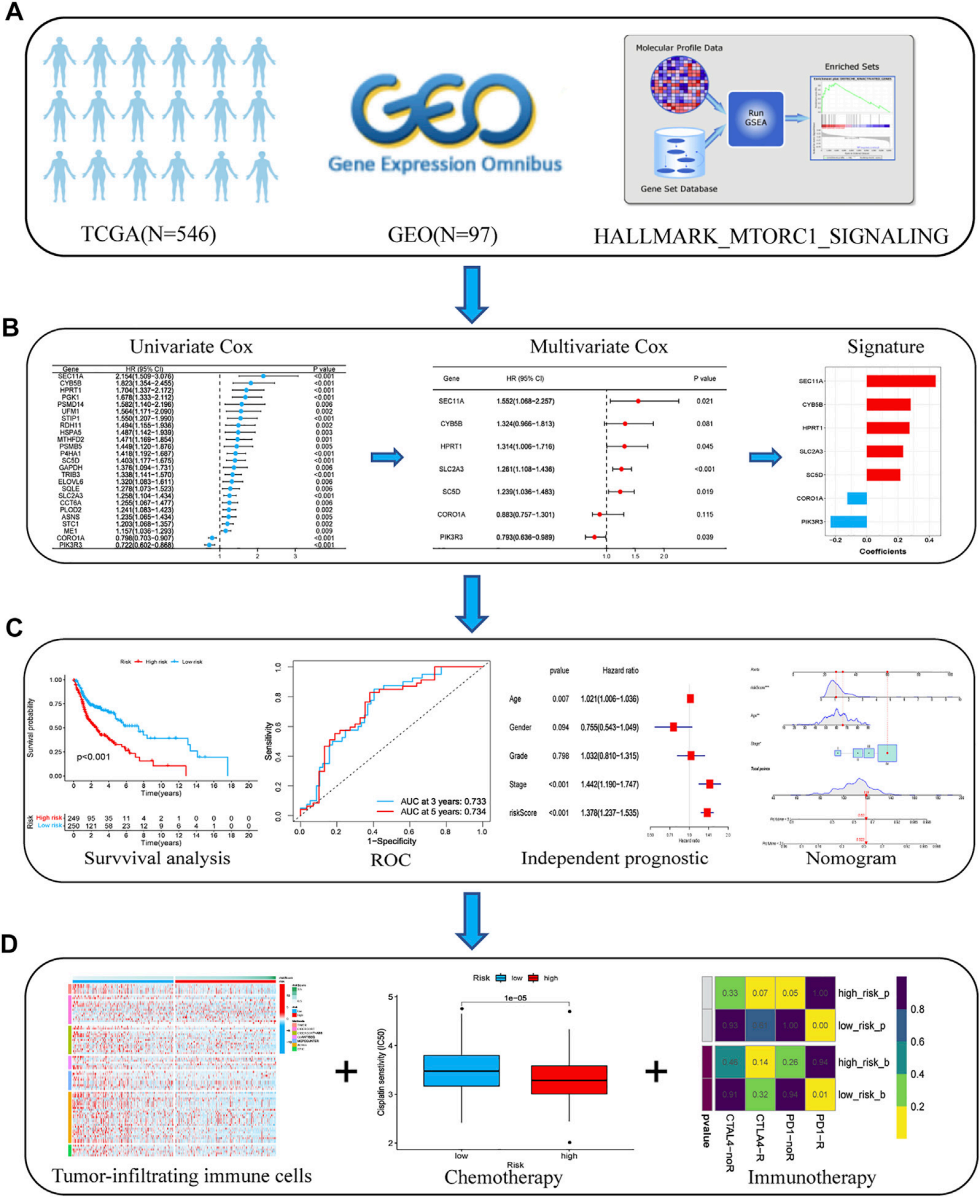
Flow chart of the analyses performed in this study. **(A)** Transcriptome data and corresponding clinical information from TCGA and GEO databases were downloaded. **(B)** Univariate and multivariate Cox regression analyses were performed to select prognostic genes and build an optimal prognostic signature. **(C)** K–M survival curves and the log-rank test were used to analyze the differences in survival times between the low- and high-risk patients. An ROC curve was plotted to investigate the prediction accuracy of the risk model. The nomogram prediction model was constructed based on the risk score and independent clinical factors, including age and AJCC stage. **(D)** We compared the infiltration status of immunocytes between the low- and high-risk groups using the presently acknowledged algorithms. Chemotherapeutic and immunotherapeutic sensitivities of patients with HNSCC were calculated and compared between the high- and low-risk groups. Abbreviations: AJCC, American Joint Committee on Cancer; GEO, Gene Expression Omnibus; HNSCC, head and neck squamous cell carcinoma; K–M, Kaplan–Meier; ROC, receiver operating characteristic; TCGA, The Cancer Genome Atlas.

The main workflow of our study is shown in Figure 1. We retrieved the HALLMARK_MTORC1_SIGNALING gene set from the Molecular Signature Database. The results of the GSEA showed that the gene set was significantly enriched in HNSCC tumor samples (normalized enrichment score (NES) = 2.01, false discovery rate (FDR) = 0.009; Supplementary Figure S1). The expression matrix of the 187 genes common to this gene set was then extracted from TCGA and GSE41613 cohort data for the subsequent analyses.

### Identification of the Seven Gene Signature

**FIGURE 2:**
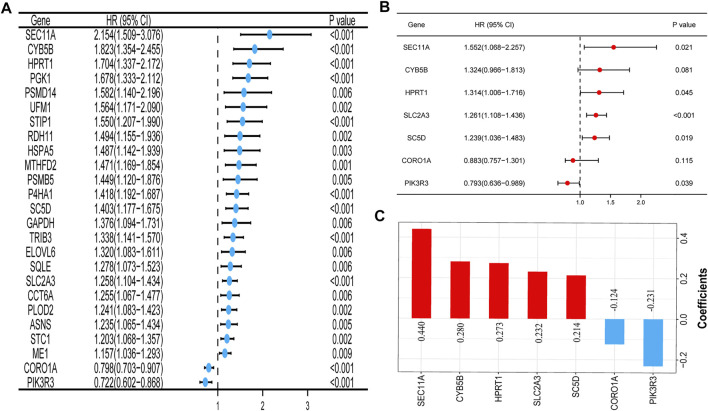
Construction of the prognostic signature of the mTOR signaling pathway–related genes in TCGA cohort. **(A)** Forest plot of the univariate Cox regression analysis of 25 prognosis-related genes. **(B)** Seven genes identified by the multivariate Cox regression analysis were used to construct a prognostic signature. **(C)** Coefficients of the seven genes included in the signature. Abbreviations: mTOR, mammalian target of rapamycin; TCGA, The Cancer Genome Atlas.

**FIGURE 3:**
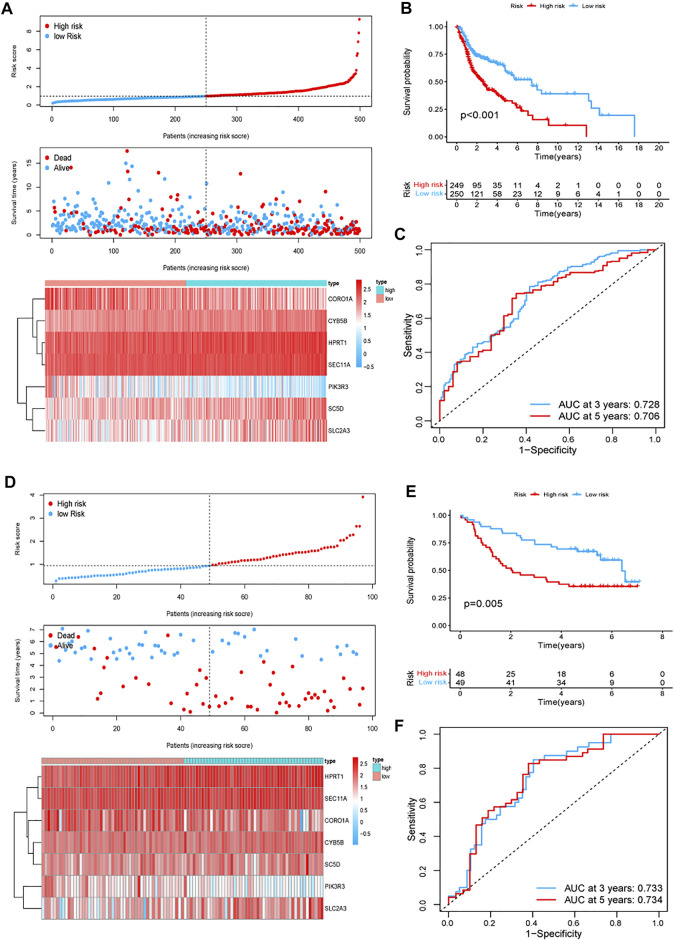
Prognostic signature analysis of patients with HNSCC in TCGA and GEO GSE41613 cohorts. **(A,D)** Distributions of the risk score, survival status, and the expression heatmap of the seven identified genes in the high- and low-risk groups in TCGA **(A)** and GEO GSE41613 **(D)** cohorts. **(B,E)** K–M curves for the two subgroups in TCGA **(B)** and GEO GSE41613 **(E)** cohorts. **(C,F)** ROC curve analysis for predicting the survival according to the risk score in TCGA **(C)** and GEO GSE41613 **(F)** cohorts. Abbreviations: GEO GSE41613, Gene Expression Omnibus GSE41613 cohort; HNSCC, head and neck squamous cell carcinoma; TCGA, The Cancer Genome Atlas.

The univariate Cox regression analysis in TCGA cohort showed that a total of 25 genes had a statistically significant correlation with the OS (*p* < 0.01, Figure 2A). These 25 genes were then included in the subsequent multivariate regression analysis, which resulted in the identification of seven genes that would be used to construct the model (Figure 2B). A risk score formula based on the regression coefficients and expression levels of the seven genes was used to determine the risk score for each patient (Figure 2C). The formula was as follows: risk score = (0.440) × expression level of SEC11A + (0.280) × expression level of CYB5B + (0.273) × expression level of HPRT1 + (0.232) × expression level of SLC2A3 + (0.214) × expression level of SC5D + (−0.124) × expression level of CORO1A+ (−0.231) × expression level of PIK3R3. According to this formula, the total risk score of all the patients was calculated, and then based on the median value, 499 patients were divided into two subgroups. For the training set, the distribution of the risk scores, each patient’s survival status, and the expression patterns of these seven risk genes in the two risk groups are presented in Figure 3A. The K–M survival analysis revealed that the OS was worse in the high-risk group than in the low-risk group (*p* < 0.001, Figure 3B), indicating that the risk score played an important role in prognostic prediction for patients with HNSCC. In the training group, the area under the curve (AUC) at 3 years was 0.728, and the 5‐year AUC was 0.706, which indicated a powerful prediction accuracy (Figure 3C).

### Validation of the Gene Signature Based on Data From the GSE41613 Cohort

To evaluate the reliability and robustness of the gene signature, we conducted validation tests using data from the GSE41613 cohort (test dataset). The risk score for the population in the validation cohort was calculated using the formula constructed using TCGA dataset. The patients with HNSCC in the GSE41613 cohort were categorized into different high- and low-risk groups based on the median value. Ultimately, 48 patients were allocated to the high-risk group and 49 were assigned to the low-risk group. Figure 3D shows the distribution of the risk scores, each patient’s survival status, and the expression patterns of the seven risk genes in the 97 patients. The K–M survival plots revealed that the high-risk group exhibited a poorer survival rate (*p* = 0.005, Figure 3E). The AUC at 3 years was 0.733, and the 5-year AUC was 0.734 (Figure 3F). These results demonstrate the robustness and stability of our gene signature.

### Landscape of Genetic Variance of the 25 Prognosis-Related Genes in HNSCC

**FIGURE 4:**
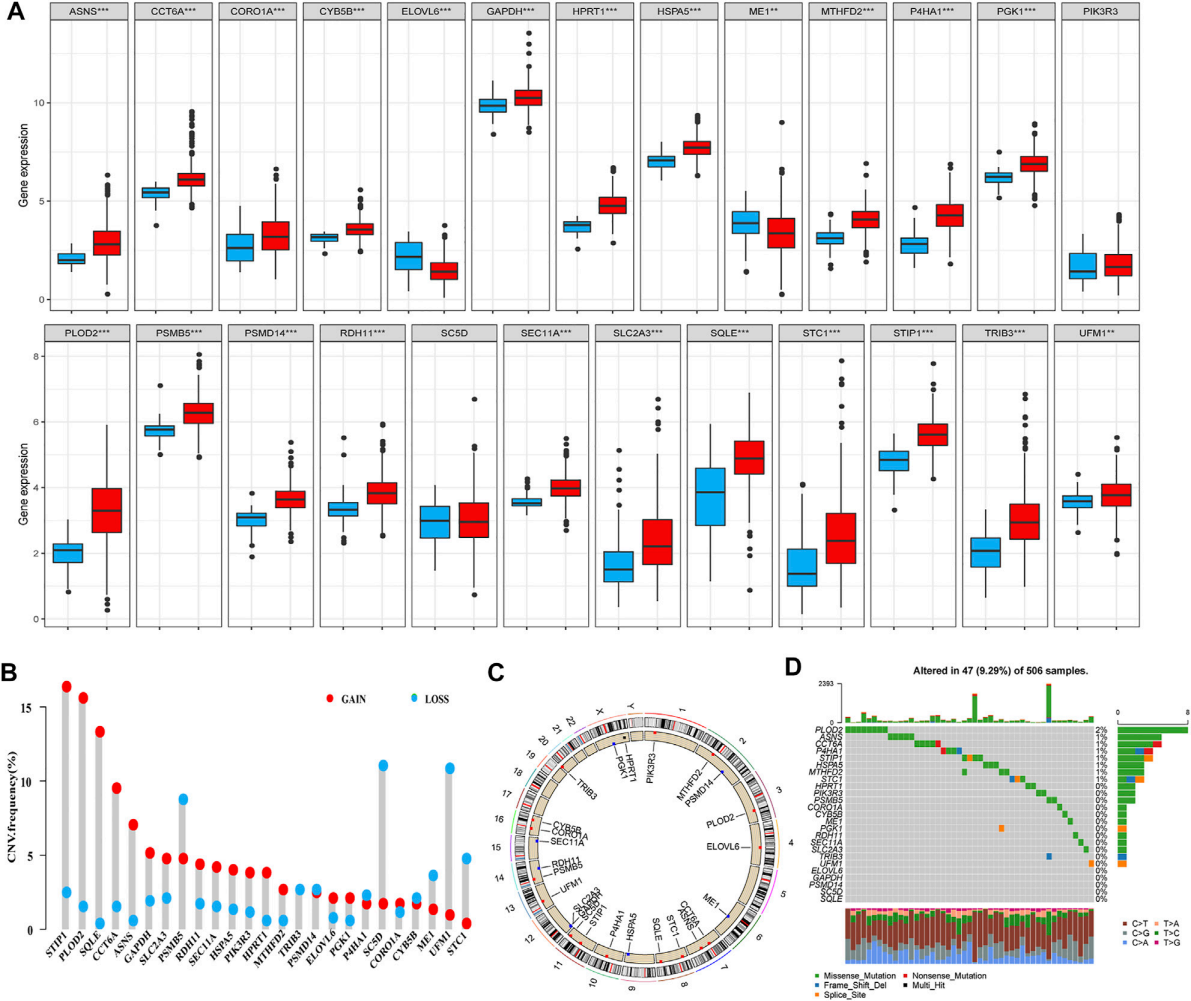
Landscape of genetic and expression variations of 25 prognostic-related genes identified through the univariate Cox regression analysis. **(A)** Expression levels of the 25 genes in HNSCC (red) and normal (dew grass color) tissues. **(B)** CNV frequency of the 25 genes in TCGA cohort. The height of the column represents the alteration frequency. CNV gain, red; CNV loss, dew grass color. **(C)** Locations of the CNV alterations of the 25 genes on 23 chromosomes in TCGA cohort. **(D)** Mutation frequency and classification of the 25 genes in HNSCC (***p < 0.001 and **p < 0.01). Abbreviations: CNV, copy number variation; HNSCC, head and neck squamous cell carcinoma; TCGA, The Cancer Genome Atlas.

We first investigated the expression levels of the identified genes in HNSCC and normal tissues among patients with HNSCC in TCGA database. Overall, 25 genes were either up- or downregulated in those with HNSCC. More specifically, in patients with HNSCC, the expression levels of CCT6A, CORO1A, CYB5B, GAPDH, HPRT1, HSPA5, MTHFD2, P4HA1, PGK1, PLOD2, PSMB5, PSMD14, RDH11, SEC11A, SLC2A3, SQLE, STC1, STIP1, TRIB3, and UFM1 were higher in tumors than in normal tissues, whereas ELOVL6 and ME1 were more highly expressed in normal tissues than in tumors (Figure 4A, *p* < 0.01). We then analyzed the incidence of CNV and somatic mutations of these genes in patients with HNSCC, which revealed frequent CNV alterations in these genes, with a higher proportion of copy number gains than losses. More than half of the 25 prognosis-related genes exhibited significant copy number gains, along with widespread CNV deletion frequencies of PSMB5, SC5D, UFM1, and STC1 (Figure 4B). Figure 4C shows the locations of the CNV alterations of these genes on chromosomes. Gene mutations were found in 47 (9.29%) of the 506 samples. Indeed, we found that PLOD2 was the most frequently mutated gene in patients with HNSCC, followed by ASNS, CCT6A, P4HA1, STIP1, HSPA5, MTHFD2, and STC1; the other 17 genes showed no mutations or mutations in less than 1% of samples. The missense mutation ranked as the top variant classification, and C > T was the most common single-nucleotide variant (SNV) class (Figure 4D).

### Prognostic Value of the Seven Genes Used to Construct the Signature

**FIGURE 5:**
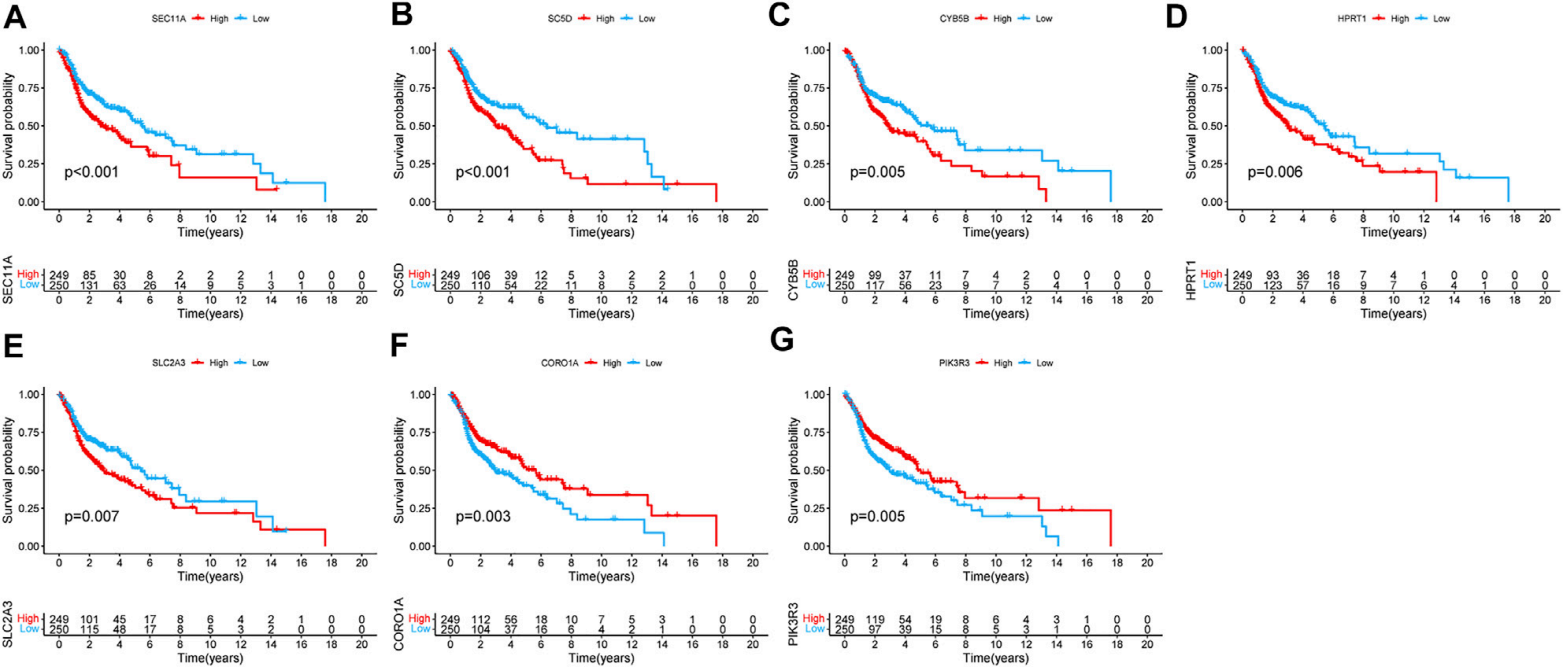
Kaplan–Meier survival analysis of the seven genes in TCGA cohort. **(A)** SEC11A, **(B)** SC5D, **(C)** CYB5B, **(D)** HPRT1, **(E)** SLC2A3, **(F)** CORO1A, and **(G)** PIK3R3.

The results of the K–M survival analysis demonstrated that all identified genes had a major impact on the clinical outcome of patients with HNSCC in TCGA database (*SEC11A*: *p* < 0.001; *SC5D*: *p* < 0.001; *CYB5B*: *p* = 0.005; *HPRT1*: *p* = 0.006; *SLC2A3*: *p* = 0.007; *CORO1A*: *p* = 0.003; *PIK3R3*: *p* = 0.005). The K–M curves showed that patients with HNSCC who had high expression levels of SEC11A, SC5D, CYB5B, HPRT1, and SLC2A3 had lower survival rates, whereas those with HNSCC who had low expression levels of CORO1A and PIK3R3 had lower survival rates (Figures 5A–G).

### Independent Prognostic Analysis and the Construction and Validation of the Nomogram

**FIGURE 6:**
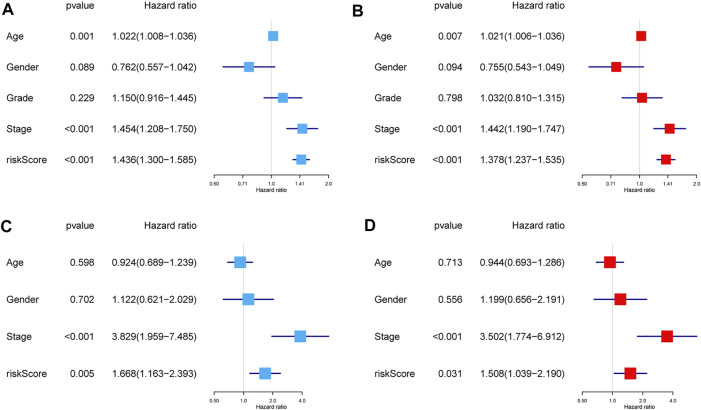
Independent prognostic factors for the OS in patients with HNSCC. Univariate and multivariate Cox regression analyses were performed using TCGA dataset **(A,B)** and GEO dataset **(C,D)**. Abbreviations: GEO, Gene Expression Omnibus; HNSCC, head and neck squamous cell carcinoma; OS, overall survival; TCGA, The Cancer Genome Atlas.

The results of the univariate Cox regression analysis in the training group revealed that age (HR = 1.022, 95% CI: 1.008–1.036), AJCC stage (HR = 1.454, 95% CI: 1.208–1.750), and risk score (HR = 1.436, 95% CI: 1.300–1.585) were related to significantly lower OS (Figure 6A). Similarly, the multivariate Cox regression analysis also revealed that age (HR = 1.021, 95% CI: 1.006–1.036), AJCC stage (HR = 1.442, 95% CI: 1.190–1.747), and risk score (HR = 1.378, 95% CI: 1.237–1.535) were associated with significantly worse OS (Figure 6B). For the test group, the results revealed that the AJCC stage (univariate: HR = 3.829, 95% CI: 1.959–7.485; multivariate: HR = 3.502, 95% CI: 1.774–6.912) and risk score (univariate: HR = 1.668, 95% CI: 1.163–2.393; multivariate: HR = 1.508, 95% CI: 1.039–2.190) were associated with worse OS among patients with HNSCC (Figures 6C, D). Collectively, these data revealed that the gene signature was an independent prognostic factor for HNSCC after adjustment for confounding factors (*p* < 0.05).

**FIGURE 7:**
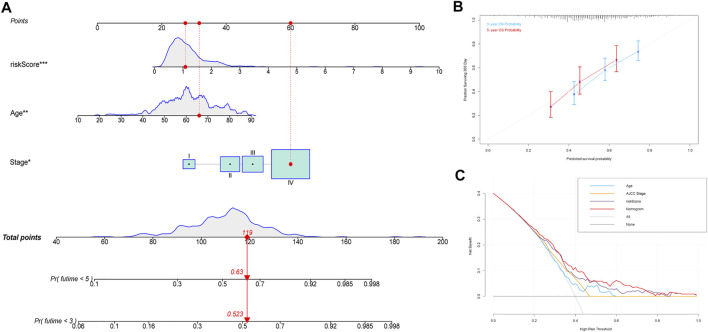
Construction of the predictive nomogram. **(A)** Nomogram predicting the OS probability for patients with HNSCC. **(B)** Calibration curves of the nomogram for predicting the OS at 3 and 5 years. **(C)** DCA analysis showing the performance of the nomogram. Abbreviations: HNSCC, head and neck squamous cell carcinoma; OS, overall survival.

Subsequently, a nomogram based on the significant clinical parameters (age and AJCC stage) and risk score was constructed to forecast survival probability in patients with HNSCC in TCGA cohort. The overall score comprises the points for each included variable and can be used to forecast survival probability at 3 and 5 years (Figure 7A). The calibration plots showed that the nomogram performed well in comparison with an ideal model, indicating that the compound nomogram has excellent reliability and veracity (Figure 7B). The clinical practicability of the model was assessed through DCA. Compared with the performance of three other models based on a single factor (AJCC stage only or age only or signature only), the hybrid nomogram based on age, AJCC stage, and the multigene signature can achieve higher net benefits (Figure 7C).

### Differences in Tumor Immune Infiltrating Cells Between the Two Risk Groups

As shown in Supplementary Figure S2, the heatmap demonstrates the levels of immune cell infiltration in the two risk groups based on seven algorithms. The infiltration levels of immune cells such as resting mast cells, resting natural killer (NK) cells, and M0 macrophages were all lower in the low‐risk group, whereas the levels of B cells and CD8^+^ T cells were lower in the high-risk group. Our findings suggest that the immune microenvironment differed between patients in the two risk groups, which may have implications for individualized treatment.

### Prediction of Patient Response to Chemotherapy and Immunotherapy in the Two Risk Groups

**FIGURE 8:**
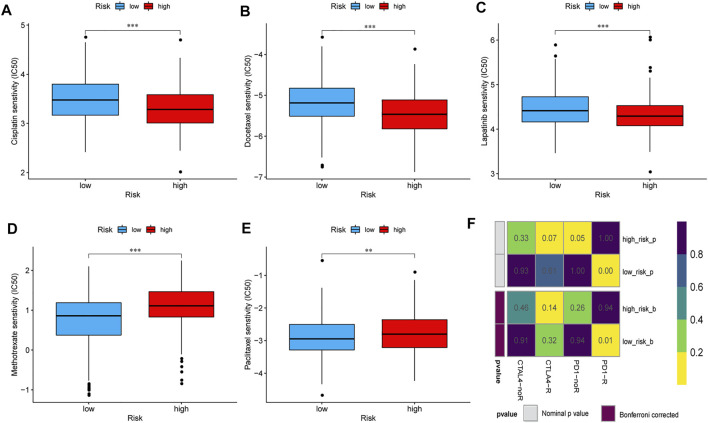
Sensitivities of patients in the high- and low-risk groups to chemotherapy and immunotherapy. The differential chemotherapeutical sensitivities to cisplatin **(A)**, docetaxel **(B)**, lapatinib **(C)**, methotrexate **(D)**, and paclitaxel **(E)** in the two risk groups. **(F)** Sensitivity to PD1 and CTLA4 inhibitors of patients in the low- and high-risk groups. Abbreviations: CTLA4, cytotoxic T lymphocyte-associated protein 4; PD1, programmed cell death 1.

This study evaluated and compared the response to treatment with cisplatin, docetaxel, lapatinib, methotrexate, and paclitaxel between the high- and low-risk groups. The IC_50_ of each patient was estimated, and the results showed statistically significant differences between the low- and high-risk groups for all five chemotherapeutic agents. As shown in the box plots, patients with a high risk score had lower IC_50_ values than those with a low risk score, which indicated that the high-risk group responded better to treatment with cisplatin, docetaxel, and lapatinib (Figures 8A–C), whereas the low-risk group was more sensitive to treatment with methotrexate and paclitaxel (Figures 8D, E). Recent studies have reported that treatment with anti–PD-1 and anti–CTLA-4 antibodies is an effective therapeutic option for patients with HNSCC (Italiano et al., 2021; Mao et al., 2021; Qin et al., 2021). The challenge of immunotherapy is that the response to a particular immunotherapeutic treatment varies among patients and can be hard to predict (Konstorum et al., 2017). Therefore, we assessed the therapeutic response of patients with HNSCC to PD-1 and CTLA-4 inhibitors. The results suggested that compared with those in the high-risk group, the low-risk group showed more promising responses to anti–PD-1 therapy (nominal *p* < *0.01*, Bonferroni corrected *p* = 0.01, Figure 8F).

### Expression of SEC11A and CYB5B Is Upregulated in HNSCC

**FIGURE 9:**
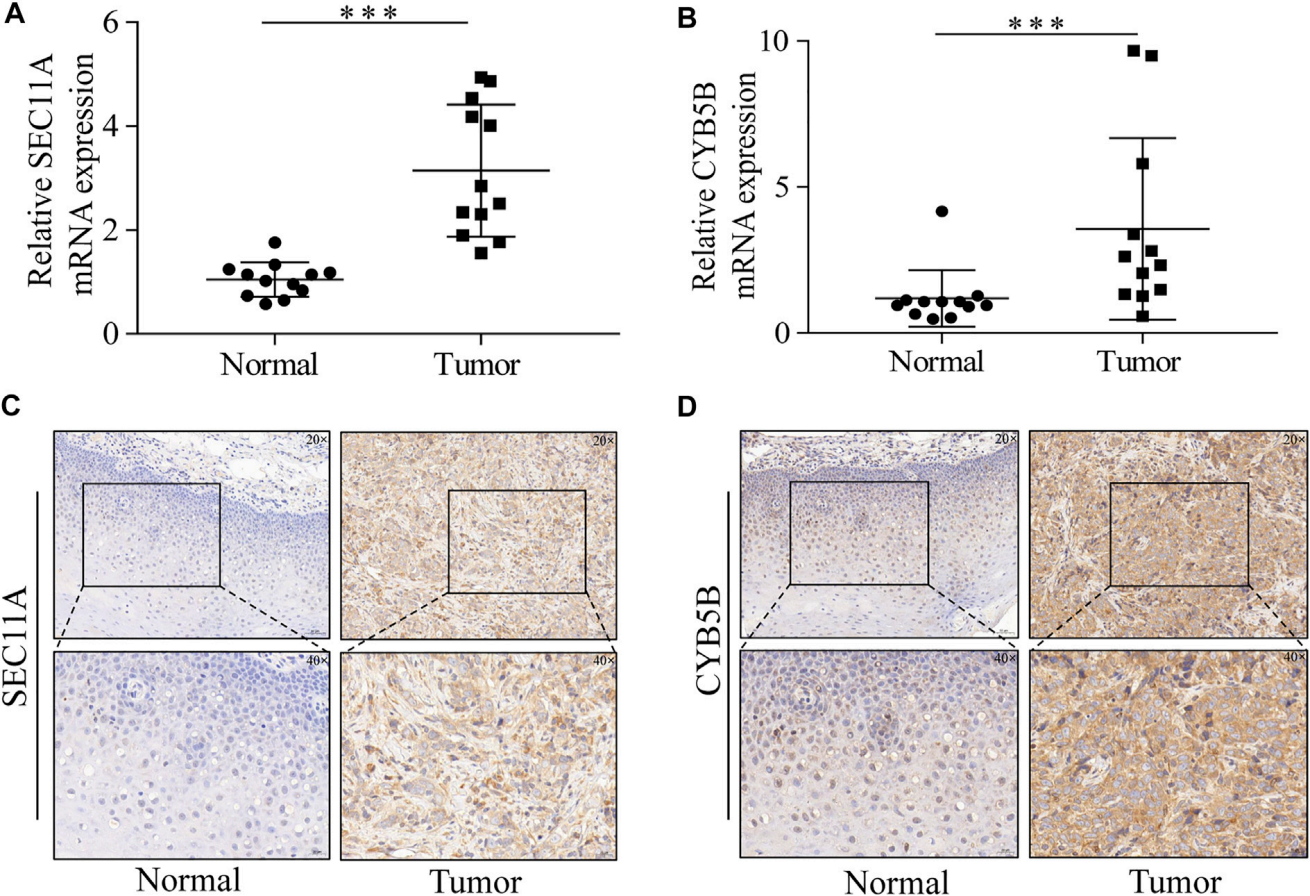
SEC11A and CYB5B expression is upregulated in HNSCC. **(A)** qRT-PCR analysis of SEC11A mRNA levels in tumor and adjacent normal tissues. **(B)** qRT-PCR analysis of CYB5B mRNA levels in tumor and adjacent normal tissues. **(C)** IHC analysis of SEC11A protein levels in tumor and adjacent normal tissues. **(D)** IHC analysis of CYB5B protein levels in tumor and adjacent normal tissues. ***, *p* < 0.001. Abbreviations: CYB5B, cytochrome B5 type B; HNSCC, head and neck squamous cell carcinoma; IHC, immunohistochemistry; mRNA, messenger ribonucleic acid; qRT-PCR, quantitative real-time polymerase chain reaction; SEC11A, signal peptidase complex catalytic subunit.

For the purpose of detecting the difference in the mRNA expression of seven signature genes, we conducted a qPCR to detect 12 pairs of cancer and normal tissues. The mRNA expression levels of SEC11A and CYB5B in tumors were significantly higher than those in normal tissues. However, no obvious difference was observed for HPRT1, SLC2A3, SC5D, CORO1A, and PIK3R3 mRNA expression levels between HNSCC tissues and adjacent normal tissues (Supplementary Figure S3). We performed further IHC experiments to quantify the levels of the proteins encoded by the two genes for which the qRT-PCR results were significant. The protein levels of SEC11A and CYB5B were significantly increased in cancer tissues (Figure 9).

## Discussion

Genetic factors play a vital role in the tumorigenesis and progression of HNSCC. Signatures based on certain features such as glycolysis (Chen et al., 2021) and immunity (Ma et al., 2020) have been discovered and may be useful for predicting the prognosis in patients with cancer. Compared with the mTORC1, little is known about the mechanisms regulating the mTORC2 and its biological function in the cell. In the present study, we constructed a novel gene signature capable of predicting the prognosis of patients with HNSCC based on specific genes related to the mTORC1 signaling pathway. A previous study showed that the phosphoinositide 3-kinase/Akt/mTOR pathway is frequently overactivated in more than 80% of individuals with HNSCC (Iglesias-Bartolome et al., 2013). Thus, the search for prognostic genes associated with the mTORC1 signaling pathway is important for guiding further research into therapeutic targets for patients with HNSCC.

In our study, a univariate Cox regression analysis was first used to identify 25 mTORC1 signaling pathway–related genes that could have important prognostic value in HNSCC. Subsequently, based on the multivariate Cox regression analysis, we identified seven key genes for constructing the signature. A worse prognosis was observed in patients with HNSCC who exhibited high levels of expression of SEC11A, SC5D, CYB5B, HPRT1, and SLC2A3 and low levels of expression of CORO1A and PIK3R3. Furthermore, we conducted qRT-PCR and IHC analyses and concluded that the mRNA and protein expression levels of SEC11A and CYB5B were higher in HNSCC tissues than in adjacent normal tissues. Among the genes included in the signature, five were found to be closely associated with HNSCC in previous studies. For example, Yao et al. (2021) found that SPC18 encoded by SEC11A is upregulated in tongue squamous cell carcinoma, and miR-873-5p may promote tumor cell apoptosis by targeting SEC11A. A recent study suggests that the overexpression of HPRT1 is correlated with the poor clinical outcome of HNSCC and can be used as a meaningful prognostic marker and therapeutic target (Ahmadi et al., 2021). Moreover, HPRT may promote the epithelial–mesenchymal transition (EMT) process and cell proliferation through direct interaction with STAT3 in HNSCC (Wang et al., 2021). Similarly, SLC2A3, which is related to glycolysis, can be used as a marker for the invasion of laryngeal squamous cell carcinoma (Starska et al., 2015). Song et al. (2019) identified 16 hub genes involved in the tumorigenesis of HNSCC by using the weighted gene coexpression network analysis, including CORO1A, and validated these genes at the transcription and translation levels. Moreover, a study showed that miR-367 can suppress the proliferation and invasion of oral squamous cell carcinoma by regulating PIK3R3 (Sun and Feng, 2020). However, the relationship between CYB5B and SC5D and HNSCC had not been clarified before, and further studies are expected to verify their prognostic value.

It is well known that most malignancies are heterogenous, and HNSCC is no exception. Significant investment to fund the study of tumor heterogeneity would undoubtedly be one of the most effective ways in improving patient survival. Prognostic models based on gene expression levels can, to some extent, compensate for the shortcomings of AJCC staging in predicting patient prognosis. The signature developed in this study exhibited strong performance in predicting the prognosis of patients with HNSCC, with the DCA results showing that the net benefit of the risk score is greater than that of the AJCC stage, and the predictive performance of the nomogram is greatly enhanced when both were used in combination with age.

Resection, radiotherapy, and systemic therapy have been established as effective treatment modalities for HNSCC (Johnson et al., 2020). Neoadjuvant chemotherapy, also known as primary chemotherapy, is widely used to reduce tumor bulk and convert unresectable tumors into resectable ones. Single drugs that are efficacious in the treatment of HNSCC include docetaxel, methotrexate, cisplatin, 5-fluorouracil (5-FU), and paclitaxel (Specenier and Vermorken, 2018). In addition, a recent phase II clinical trial demonstrated that a novel induction regimen that included the administration of lapatinib before transoral surgery was feasible, and positive results were achieved in patients with HNSCC (Hackman et al., 2020). In this study, we assessed the chemotherapeutic sensitivity to five drugs in patients with HNSCC and compared it between the two risk groups by using IC_50_ values as an evaluation index. The patients in the high-risk group exhibited higher chemotherapeutic sensitivity to cisplatin, docetaxel, and lapatinib, whereas patients in the low-risk group showed higher sensitivity to methotrexate and paclitaxel.

The activity of mTORC1 is critical for maintaining immune cell function, and the inhibition of mTORC1 can significantly alter or completely ablate the immune response (Abraham and Wiederrecht, 1996). Wang et al. (2020b) found that mTORC1 was a promising target for tumor blood vessel normalization, which could reinforce antitumor immunity. Tumor immune cell infiltration reflects the immune microenvironment around the tumor tissues and can impact the response to immunotherapy (Liu et al., 2020). In squamous cell carcinomas, low immune infiltration was found to be correlated with worse prognosis of patients (Balermpas et al., 2014). Moreover, immune checkpoint (PD-1 and CTLA-4) blockade is gaining recognition as a paradigm for effective cancer treatment (Ribas and Wolchok, 2018). In this study, we compared the infiltration status of immunocytes between the two risk groups and assessed the therapeutic response to PD-1 and CTLA-4 inhibitors. Significant differences in the abundance of immune infiltrating cells such as quiescent mast cells, quiescent NK cells, M0 macrophages, B cells, and CD8^+^ T cells were observed between the high- and low-risk groups. This finding explains the difference in the prognosis of patients with HNSCC in terms of the changes in the immune microenvironment of tumors. Finally, the comparison of the ability to predict immunotherapy responses between both groups revealed a greater benefit for patients in the lower-risk group when receiving anti–PD-1 therapy, which could be helpful in guiding clinical treatment.

The most important contribution of this study is the development of a novel and potentially applicable tool that is not only capable of predicting the prognosis of patients with HNSCC but can also help identify individuals who would benefit most from chemotherapy and immunotherapy. However, there are still some limitations to this study. First, it involved a bioinformatics analysis of database information, and the findings need to be validated using data from multiple centers. Second, the potential mechanisms through which the seven genes in the prognostic signature are involved in the occurrence or development of HNSCC are not entirely understood, and further investigation would require a series of experiments, both *in vivo* and *in vitro*.

## Conclusion

We have developed a prognostic gene signature based on the mTORC1 signaling pathway that can be used as a helpful tool in predicting the prognosis of patients with HNSCC and their sensitivity to chemotherapy and immunotherapy.

## Data Availability

Publicly available datasets were analyzed in this study. The raw data of this study are derived from the TCGA database (https://portal.gdc.cancer.gov/) and GEO data portal (https://www.ncbi.nlm.nih.gov/geo/), which are publicly available databases.

## References

[B1] Abraham R. T. Wiederrecht G. J. (1996). Immunopharmacology of Rapamycin. Annu. Rev. Immunol. 14, 483–510. 10.1146/annurev.immunol.14.1.483 8717522

[B2] Ahmadi M. Eftekhari Kenzerki M. Akrami S. M. Pashangzadeh S. Hajiesmaeili F. Rahnavard S. (2021). Overexpression of HPRT1 Is Associated with Poor Prognosis in Head and Neck Squamous Cell Carcinoma. FEBS Open Bio 11, 2525–2540. 10.1002/2211-5463.13250 PMC8409317 34231338

[B3] Aran D. (2020). Cell-Type Enrichment Analysis of Bulk Transcriptomes Using xCell. Methods Mol. Biol. 2120, 263–276. 10.1007/978-1-0716-0327-7_19 32124326

[B4] Balermpas P. Michel Y. Wagenblast J. Seitz O. Weiss C. Rödel F. (2014). Tumour-infiltrating Lymphocytes Predict Response to Definitive Chemoradiotherapy in Head and Neck Cancer. Br. J. Cancer 110, 501–509. 10.1038/bjc.2013.640 24129245 PMC3899751

[B5] Bazzichetto C. Conciatori F. Falcone I. Ciuffreda L. (2020). Translational Landscape of mTOR Signaling in Integrating Cues between Cancer and Tumor Microenvironment. Adv. Exp. Med. Biol. 1223, 69–80. 10.1007/978-3-030-35582-1_4 32030685

[B6] Bray F. Ferlay J. Soerjomataram I. Siegel R. L. Torre L. A. Jemal A. (2018). Global Cancer Statistics 2018: GLOBOCAN Estimates of Incidence and Mortality Worldwide for 36 Cancers in 185 Countries. CA: A Cancer J. Clinicians 68, 394–424. 10.3322/caac.21492 30207593

[B7] Califano J. van der Riet P. Westra W. Nawroz H. Clayman G. Piantadosi S. (1996). Genetic Progression Model for Head and Neck Cancer: Implications for Field Cancerization. Cancer Res. 56, 2488–2492. 8653682

[B8] Caro-Vegas C. Bailey A. Bigi R. Damania B. Dittmer D. P. (2019). Targeting mTOR with MLN0128 Overcomes Rapamycin and Chemoresistant Primary Effusion Lymphoma. mBio 10. 10.1128/mBio.02871-18 PMC6381283 30782662

[B9] Chen B. Khodadoust M. S. Liu C. L. Newman A. M. Alizadeh A. A. (2018). Profiling Tumor Infiltrating Immune Cells with CIBERSORT. Methods Mol. Biol. 1711, 243–259. 10.1007/978-1-4939-7493-1_12 29344893 PMC5895181

[B10] Chen L. He X. Yi S. Liu G. Liu Y. Ling Y. (2021). Six Glycolysis-Related Genes as Prognostic Risk Markers Can Predict the Prognosis of Patients with Head and Neck Squamous Cell Carcinoma. Biomed. Res. Int. 2021, 8824195. 10.1155/2021/8824195 33628816 PMC7889344

[B11] Dienstmann R. Villacampa G. Sveen A. Mason M. J. Niedzwiecki D. Nesbakken A. (2019). Relative Contribution of Clinicopathological Variables, Genomic Markers, Transcriptomic Subtyping and Microenvironment Features for Outcome Prediction in Stage II/III Colorectal Cancer. Ann. Oncol. 30, 1622–1629. 10.1093/annonc/mdz287 31504112 PMC6857614

[B12] Ferris R. L. (2015). Immunology and Immunotherapy of Head and Neck Cancer. Jco 33, 3293–3304. 10.1200/jco.2015.61.1509 PMC4586169 26351330

[B13] Hackman T. G. Patel S. N. Deal A. M. Neil Hayes D. Chera B. S. Paul J. (2020). Novel Induction Therapy Transoral Surgery Treatment Paradigm with Risk-Adapted Adjuvant Therapy for Squamous Cell Carcinoma of the Head and Neck - Mature Clinical and Functional Outcomes. Oral Oncol. 110, 104957. 10.1016/j.oraloncology.2020.104957 32823258

[B14] He F. Chen Z. Deng W. Zhan T. Huang X. Zheng Y. (2021). Development and Validation of a Novel Ferroptosis-Related Gene Signature for Predicting Prognosis and Immune Microenvironment in Head and Neck Squamous Cell Carcinoma. Int. immunopharmacology 98, 107789. 10.1016/j.intimp.2021.107789 34130150

[B15] Iglesias-Bartolome R. Martin D. Gutkind J. S. (2013). Exploiting the Head and Neck Cancer Oncogenome: Widespread PI3K-mTOR Pathway Alterations and Novel Molecular Targets. Cancer Discov. 3, 722–725. 10.1158/2159-8290.cd-13-0239 23847349 PMC4348071

[B16] Italiano A. Cassier P. A. Lin C. C. Alanko T. Peltola K. J. Gazzah A. (2021). First-in-human Phase 1 Study of Budigalimab, an Anti-PD-1 Inhibitor, in Patients with Non-small Cell Lung Cancer and Head and Neck Squamous Cell Carcinoma. Cancer Immunol Immunother 71, 417-431. 10.1007/s00262-021-02973-w 34216247 PMC8783908

[B17] Jemal A. Bray F. Center M. M. Ferlay J. Ward E. Forman D. (2011). Global Cancer Statistics. CA: A Cancer J. Clinicians 61, 69–90. 10.3322/caac.20107 21296855

[B18] Johnson D. E. Burtness B. Leemans C. R. Lui V. W. Y. Bauman J. E. Grandis J. R. (2020). Head and Neck Squamous Cell Carcinoma. Nat. Rev. Dis. Primers 6, 92. 10.1038/s41572-020-00224-3 33243986 PMC7944998

[B19] Kokko L.-L. Hurme S. Maula S.-M. Alanen K. Grénman R. Kinnunen I. (2011). Significance of Site-specific Prognosis of Cancer Stem Cell Marker CD44 in Head and Neck Squamous-Cell Carcinoma. Oral Oncol. 47, 510–516. 10.1016/j.oraloncology.2011.03.026 21514878

[B20] Konstorum A. Vella A. T. Adler A. J. Laubenbacher R. C. (2017). Addressing Current Challenges in Cancer Immunotherapy with Mathematical and Computational Modelling. J. R. Soc. Interf. 14. 10.1098/rsif.2017.0150 PMC5493798 28659410

[B21] Li T. Fu J. Zeng Z. Cohen D. Li J. Chen Q. (2020). TIMER2.0 for Analysis of Tumor-Infiltrating Immune Cells. Nucleic Acids Res. 48, W509–W514. 10.1093/nar/gkaa407 32442275 PMC7319575

[B22] Liu R. Hu R. Zeng Y. Zhang W. Zhou H.-H. (2020). Tumour Immune Cell Infiltration and Survival after Platinum-Based Chemotherapy in High-Grade Serous Ovarian Cancer Subtypes: A Gene Expression-Based Computational Study. EBioMedicine 51, 102602. 10.1016/j.ebiom.2019.102602 31911269 PMC6948169

[B23] Liu Z. Zhang D. Liu C. Li G. Chen H. Ling H. (2021). Comprehensive Analysis of Myeloid Signature Genes in Head and Neck Squamous Cell Carcinoma to Predict the Prognosis and Immune Infiltration. Front. Immunol. 12, 659184. 10.3389/fimmu.2021.659184 33995379 PMC8116959

[B24] Ma B. Li H. Qiao J. Meng T. Yu R. (2020). Immune-related miRNA Signature Identifies Prognosis and Immune Landscape in Head and Neck Squamous Cell Carcinomas. Biosci. Rep. 40. 10.1042/BSR20201820 PMC7670576 33111959

[B25] Machiels J.-P. René Leemans C. Golusinski W. Grau C. Licitra L. Gregoire V. (2020). Squamous Cell Carcinoma of the Oral Cavity, Larynx, Oropharynx and Hypopharynx: EHNS-ESMO-ESTRO Clinical Practice Guidelines for Diagnosis, Treatment and Follow-Up. Ann. Oncol. 31, 1462–1475. 10.1016/j.annonc.2020.07.011 33239190

[B26] Mao L. Xiao Y. Yang Q.-C. Yang S.-C. Yang L.-L. Sun Z.-J. (2021). TIGIT/CD155 Blockade Enhances Anti-PD-L1 Therapy in Head and Neck Squamous Cell Carcinoma by Targeting Myeloid-Derived Suppressor Cells. Oral Oncol. 121, 105472. 10.1016/j.oraloncology.2021.105472 34333450

[B27] Meric-Bernstam F. Gonzalez-Angulo A. M. (2009). Targeting the mTOR Signaling Network for Cancer Therapy. Jco 27, 2278–2287. 10.1200/jco.2008.20.0766 PMC2738634 19332717

[B28] Mermel C. H. Schumacher S. E. Hill B. Meyerson M. L. Beroukhim R. Getz G. (2011). GISTIC2.0 Facilitates Sensitive and Confident Localization of the Targets of Focal Somatic Copy-Number Alteration in Human Cancers. Genome Biol. 12, R41. 10.1186/gb-2011-12-4-r41 21527027 PMC3218867

[B29] Mossmann D. Park S. Hall M. N. (2018). mTOR Signalling and Cellular Metabolism Are Mutual Determinants in Cancer. Nat. Rev. Cancer 18, 744–757. 10.1038/s41568-018-0074-8 30425336

[B30] Plattner C. Finotello F. Rieder D. (2020). Deconvoluting Tumor-Infiltrating Immune Cells from RNA-Seq Data Using quanTIseq. Methods Enzymol. 636, 261–285. 10.1016/bs.mie.2019.05.056 32178821

[B31] Polverini P. J. D’Silva N. J. Lei Y. L. (2018). Precision Therapy of Head and Neck Squamous Cell Carcinoma. J. Dent Res. 97, 614–621. 10.1177/0022034518769645 29649374 PMC5960884

[B32] Qin Y. Zheng X. Gao W. Wang B. Wu Y. (2021). Tumor Microenvironment and Immune-Related Therapies of Head and Neck Squamous Cell Carcinoma. Mol. Ther. - Oncolytics 20, 342–351. 10.1016/j.omto.2021.01.011 33614915 PMC7878981

[B33] Racle J. de Jonge K. Baumgaertner P. Speiser D. E. Gfeller D. (2017). Simultaneous Enumeration of Cancer and Immune Cell Types from Bulk Tumor Gene Expression Data. Elife 6. 10.7554/eLife.26476 PMC5718706 29130882

[B34] Ribas A. Wolchok J. D. (2018). Cancer Immunotherapy Using Checkpoint Blockade. Science 359, 1350–1355. 10.1126/science.aar4060 29567705 PMC7391259

[B35] Saxton R. A. Sabatini D. M. (2017). mTOR Signaling in Growth, Metabolism, and Disease. Cell 168, 960–976. 10.1016/j.cell.2017.02.004 28283069 PMC5394987

[B36] Shao Y. Wolf P. G. Guo S. Guo Y. Gaskins H. R. Zhang B. (2017). Zinc Enhances Intestinal Epithelial Barrier Function through the PI3K/AKT/mTOR Signaling Pathway in Caco-2 Cells. J. Nutr. Biochem. 43, 18–26. 10.1016/j.jnutbio.2017.01.013 28193579

[B37] Song Y. Pan Y. Liu J. (2019). The Relevance between the Immune Response-Related Gene Module and Clinical Traits in Head and Neck Squamous Cell Carcinoma. Cmar 11, 7455–7472. 10.2147/cmar.s201177 PMC6689548 31496804

[B38] Specenier P. Vermorken J. B. (2018). Optimizing Treatments for Recurrent or Metastatic Head and Neck Squamous Cell Carcinoma. Expert Rev. Anticancer Ther. 18, 901–915. 10.1080/14737140.2018.1493925 29999437

[B39] Starska K. Forma E. Jóźwiak P. Bryś M. Lewy-Trenda I. Brzezińska-Błaszczyk E. (2015). Gene and Protein Expression of Glucose Transporter 1 and Glucose Transporter 3 in Human Laryngeal Cancer-The Relationship with Regulatory Hypoxia-Inducible Factor-1α Expression, Tumor Invasiveness, and Patient Prognosis. Tumor Biol. 36, 2309–2321. 10.1007/s13277-014-2838-4 PMC4428538 25412955

[B40] Sun H. Feng X. (2020). MicroRNA-367 Directly Targets PIK3R3 to Inhibit Proliferation and Invasion of Oral Carcinoma Cells. Biosci. Rep. 40. 10.1042/BSR20193867 PMC7260354 32378714

[B41] Tamminga M. Hiltermann T. J. N. Schuuring E. Timens W. Fehrmann R. S. Groen H. J. (2020). Immune Microenvironment Composition in Non-small Cell Lung Cancer and its Association with Survival. Clin. Transl Immunol. 9, e1142. 10.1002/cti2.1142 PMC7291326 32547744

[B42] Tijink B. M. Buter J. de Bree R. Giaccone G. Lang M. S. Staab A. (2006). A Phase I Dose Escalation Study with Anti-CD44v6 Bivatuzumab Mertansine in Patients with Incurable Squamous Cell Carcinoma of the Head and Neck or Esophagus. Clin. Cancer Res. 12, 6064–6072. 10.1158/1078-0432.ccr-06-0910 17062682

[B43] Wang L. Wang Y. Han N. Wang X. Ruan M. (2021). HPRT Promotes Proliferation and Metastasis in Head and Neck Squamous Cell Carcinoma through Direct Interaction with STAT3. Exp. Cel Res. 399, 112424. 10.1016/j.yexcr.2020.112424 33340493

[B44] Wang S. Raybuck A. Shiuan E. Cho S. H. Wang Q. Brantley-Sieders D. M. (2020). Selective Inhibition of mTORC1 in Tumor Vessels Increases Antitumor Immunity. JCI Insight 5. 10.1172/jci.insight.139237 PMC7455083 32759497

[B45] Wang Z. Guo X. Gao L. Wang Y. Ma W. Xing B. (2020). Glioblastoma Cell Differentiation Trajectory Predicts the Immunotherapy Response and Overall Survival of Patients. Aging 12, 18297–18321. 10.18632/aging.103695 32957084 PMC7585071

[B46] Yao Y. Liu X. Q. Yang F. Y. Mu J. W. (2021). MiR-873-5p Modulates Progression of Tongue Squamous Cell Carcinoma via Targeting SEC11A. Oral Dis. 2021. 10.1111/odi.13830 33675129

[B47] Yu S. S. Cirillo N. (2020). The Molecular Markers of Cancer Stem Cells in Head and Neck Tumors. J. Cel Physiol 235, 65–73. 10.1002/jcp.28963 31206697

