# Supplementary material for: Comprehensive Analysis of mTORC1 Signaling Pathway–Related Genes in the Prognosis of HNSCC and the Response to Chemotherapy and Immunotherapy
Source: Front Mol Biosci. 2022 Apr 29;9:792482. doi: 10.3389/fmolb.2022.792482 (PMC9100579; doi:10.3389/fmolb.2022.792482)
Supplement: Supplementary file 1 [file Table1.docx]

| Primer | Sequence (5’-3′) |
| --- | --- |
| SEC11A | (forward)TGGAAGTGAAAGTCCGATTGTA  (reverse)AAACAACAATTTCTCCCACTCG |
| CYB5B | (forward)CTGGTGTAGATGCAAGTGAAAG  (reverse)ACCACTTTCAGGTTTAAGGTCA |
| HPRT1 | (forward)TCGAGATGTGATGAAGGAGATG  (reverse)CAGCAAAGAATTTATAGCCCCC |
| SLC2A3 | (forward)TTCAATGCTGATTGTCAACCTG  (reverse)GCATTTCAACCGACTTAGCTAC |
| SC5D | (forward)GCTCATCATACAGACCACCATA  (reverse)TGAAGGATTTTTGAATGAGCCG |
| CORO1A | (forward)CCAACATCGTCTACCTCTGTGG  (reverse)CTCACACTTGTTCACCTCCAGG |
| PIK3R3 | (forward)GAGTATGGACCGCGATGA  (reverse)TTGGCTTAGGTGGCTTTG |
| GAPDH | (forward)CTGGGCTACACTGAGCACC  (reverse)AAGTGGTCGTTGAGGGCAATG |

**Table S1 Primer sequences for qPCR**
